# Targeting Phosphatidylserine in Advanced Gastric and Gastroesophageal Junction Adenocarcinomas: A Phase 2 Trial of Bavituximab Plus Pembrolizumab with Biomarker-Correlated Outcomes

**DOI:** 10.3390/curroncol33060319

**Published:** 2026-05-28

**Authors:** Panagiotis Ntellas, Haeseong Park, Kerry Culm, Jeeyun Lee, Hagop Youssoufian, Colleen Mockbee, Mark Uhlik, Laura Benjamin, Ian Chau

**Affiliations:** 1 Department of Medicine, The Royal Marsden Hospital, Downs Road, Sutton SM25PT, UK; 2 Division of Oncology, Washington University School of Medicine, St. Louis, MO 63110, USA; 3 OncXerna Therapeutics, Waltham, MA 02451, USA; 4 Department of Medicine, Samsung Medical Center, Sungkyunkwan University School of Medicine, Seoul 06351, Republic of Korea; 5 Department of Medicine, Brown University Health, Providence, RI 02912, USA

**Keywords:** gastroesophageal adenocarcinoma, gastric adenocarcinoma, gastroesophageal junction adenocarcinomas, GOJ, pembrolizumab, immunotherapy, immunomodulation, biomarkers, tumour microenvironment, TME, phosphatidylserine, bavituximab, immune checkpoint inhibitors

## Abstract

Advanced gastric and gastroesophageal junction (gastric/GOJ) adenocarcinomas have poor outcomes, and the benefit of immune checkpoint inhibitors (CPIs) remains limited. Bavituximab is a phosphatidylserine-targeting monoclonal antibody that modulates the immune and vascular tumour microenvironment. This Phase 2, open-label, multinational study evaluated bavituximab plus pembrolizumab in previously treated advanced gastric/GOJ. Patients were stratified into CPI-naïve (*n* = 61) and CPI-relapse (*n* = 19) cohorts. Bavituximab (3 mg/kg weekly) was combined with pembrolizumab (200 mg every 3 weeks) until disease progression or unacceptable toxicity. Primary endpoints included the safety and objective response rate (ORR) per RECIST 1.1. Secondary endpoints included the disease control rate (DCR), progression-free survival, overall survival, and exploratory biomarker analyses using the Xerna TME Panel and the neutrophil-to-lymphocyte ratio (NLR). No dose-limiting toxicities were observed. The most common treatment-emergent adverse events were fatigue (32.5%), constipation (31.3%), and decreased appetite (31.3%). The ORR was 13.1% in CPI-naïve and 5.3% in CPI-relapse patients; the median duration of response was 12.5 months in the CPI-naïve cohort. The DCR was 39.3% and 52.6% respectively. Higher ORRs were observed in patients with immune-high (B+) subtypes (21.9%), NLR < 4 (17.9%) and B+/NLR < 4 (33%). Bavituximab plus pembrolizumab was well tolerated but showed modest activity, with greater benefit in biomarker-defined subgroups, supporting the biomarker-driven development of tumour microenvironment-targeting combinations in advanced gastric/GOJ adenocarcinomas. NCT04099641.

## Simple Summary

This study looked at a new treatment for advanced cancers of the stomach and oesophagus, where current options often work poorly. Researchers combined pembrolizumab, an immunotherapy, with bavituximab, a drug designed to change the tumour environment and help the immune system work better. In 80 patients whose cancer had already been treated with other medicines, the combination was generally safe and side effects were manageable. The treatment helped only a modest number of patients overall, but when it did work, the benefit could last a long time. Patients whose tumours and blood tests suggested a more “immune-active” cancer were more likely to respond. Although the treatment did not show clear benefit for all patients, the results support further research into combining tumour microenvironment-targeting drugs with immunotherapy, using biomarkers to identify those most likely to benefit.

## 1. Introduction

Gastroesophageal carcinomas are a global health challenge, accounting for approximately 1,479,066 new cases and 1,104,982 deaths in 2022 [1]. These malignancies continue to have poor outcomes. The incidence-to-mortality ratio reflects their aggressive nature, with gastric cancer having a ratio of approximately 1 to 0.68, meaning that around 68% of those diagnosed will ultimately succumb to the disease. The situation is worse for oesophageal tumours, with a ratio of 1 to 0.87 [1]. Although combined modality treatment with surgical resection offers a chance for cure, the prognosis has notoriously been unfavourable in the advanced setting, with a 5-year overall survival (OS) of less than 20% [2,3].

Recently, the incorporation of immune checkpoint inhibitors (CPIs) with cytotoxic chemotherapy has improved survival outcomes for patients with gastroesophageal adenocarcinomas (GEAs) [2,4,5]. Based on the Checkmate-649, KEYNOTE-859 and Rationale 305 trials, the PD-1 inhibitors nivolumab, pembrolizumab and tislelizumab, respectively, have provided superior clinical benefit when used in combination with chemotherapy as a first-line treatment for advanced GEAs [6,7,8]. Although the survival benefit is most remarkable in patients with mismatch repair (MMR) deficiency, which represents a relatively small subset of cases, the majority of patients with microsatellite-stable (MSS) tumours still experience modest improvements dependent on their PD-L1 status [4,9]. These outcomes highlight both the potential and limitations of current immunotherapy approaches in addressing the needs of patients with GEAs, with a growing body of evidence underscoring the significant role of the tumour microenvironment (TME) in influencing treatment efficacy [10,11].

Bavituximab is an unconjugated, human–mouse chimeric G1 immunoglobulin (IgG1) designed to target phosphatidylserine (PS), an anionic phospholipid typically found on the inner leaflet of the plasma membrane [12,13,14]. Under normal conditions, ATP-dependent aminophospholipid translocases help restrict PS to the cytosolic side of the membrane [13,15]. However, during apoptosis, PS is translocated to the outer leaflet of the membrane due to the activities of translocases, scramblases and ABC floppases [16]. This externalized PS acts as an “eat me” signal leading to the clearance of apoptotic cells by macrophages, preventing unwanted inflammation and maintaining tissue tolerance [13,17,18]. Various pathologic processes, including cellular stress within the tumour microenvironment, can also disrupt this asymmetry, granting bavituximab antitumor specificity [15,19]. Additionally, PS exposure on endothelial cells within the tumour vasculature, on tumour-derived macrovesicles and on some tumour cells promotes an immunosuppressive phenotype characterized by reducing dendritic cell maturation and antigen presentation, down-regulation of TNF-α and IL-1β secretion, and upregulation of TGF-β and IL-10 [15,18,20,21].

Beyond serving as a marker of membrane stress, externalized PS functions as a dominant immunoregulatory signal within the tumour microenvironment [22]. In cancer, PS exposed on tumour cells, endothelial cells, and tumour-derived vesicles engages PS-sensing receptors, including members of the TIM and TAM receptor families, on macrophages, dendritic cells, and other myeloid populations, promoting efferocytosis-like signalling [22,23]. This process favours M2-like macrophage polarization, expansion of myeloid-derived suppressor cells, impaired dendritic cell maturation, and downstream T-cell dysfunction or exhaustion, thereby promoting local immune suppression and creating conditions that may limit responsiveness to PD-1 blockade [20,22,23,24]. In gastroesophageal malignancies, a suppressive tumour microenvironment enriched for tumour-associated macrophages and myeloid-derived suppressor cells is common, with these myeloid populations contributing to impaired dendritic-cell function, reduced antigen presentation, T-cell dysfunction, and resistance to effective antitumour immunity [11,25,26]; PS targeting may therefore complement pembrolizumab by interrupting an upstream innate immune checkpoint, reprogramming myeloid signalling, enhancing antigen presentation, and permitting more robust antitumour T-cell responses [22,23,27].

Bavituximab forms a complex with PS and β2 glycoprotein 1 (β2GP1), triggering multiple antitumor mechanisms [12]. Bavituximab can act as a vascular-targeting agent, leading to the destruction of tumour vessels by recruiting host immune functions, such as antibody-dependent cellular cytotoxicity (ADCC) [12,28,29]. Importantly, the bavituximab–β2GP1/PS complex can exert immunomodulatory effects by repolarizing M2 macrophages toward an M1 phenotype, producing pro-inflammatory cytokines such as TNF-α, IFN-g, IL-12 and IL-1β, promoting dendritic cell maturation, and stimulating tumour-specific cytotoxic T lymphocyte responses [17,20,21,30,31].

The optimal biological dose of bavituximab has been previously identified as 3 mg/kg weekly, demonstrating tolerability as both monotherapy and in combination with chemotherapy, with the most common adverse events as a single agent being fatigue, nausea, aPTT prolongation, pyrexia, rash and dyspnoea [12,32,33]. Its immune-stimulating effects suggest that bavituximab may also enhance the efficacy of CPIs, with preclinical data indicating a synergistic effect when used in combination with PD-1 inhibitors [33,34,35]. Additionally, in the SUNRISE Phase 3 trial, a post-hoc analysis revealed that lung cancer patients treated with bavituximab and docetaxel and subsequently receiving immunotherapy experienced improved OS [30], suggesting a durable priming function associated with bavituximab. We conducted this Phase 2 study to evaluate the safety, tolerability, and antitumor activity of bavituximab in combination with the PD-1 inhibitor pembrolizumab in previously treated patients with advanced gastric/GOJ cancer while also exploring the prognostic value of an RNA-expression immune signature using the Xerna TME panel.

## 2. Materials and Methods

### 2.1. Study Design

This is a Phase 2, open-label, non-randomized, two-cohort study, conducted in 21 centres in the US, UK, South Korea and Taiwan. This study included an initial safety analysis to assess the safety and tolerability of bavituximab in combination with pembrolizumab in patients with advanced gastric or GOJ adenocarcinoma who have either progressed on standard chemotherapy and were naïve to CPI therapy (Group 1) or have progressed following treatment with CPI either alone or in combination with chemotherapy (Group 2). This study was conducted in compliance with the protocol, and in accordance with the ICH GCP guidelines, FDA regulatory requirements and other regulatory authorities. All patients provided written informed consent prior to enrolment. This study is registered on ClinicalTrials.gov (NCT04099641).

Eligible individuals were aged 18 years or older (20 years or older in South Korea and Taiwan) with a histologically confirmed diagnosis of gastric or GOJ adenocarcinoma that was metastatic or unresectable at the time of enrolment and progressed on or after at least one prior regimen for advanced disease. In the Group 1 (CPI-naïve), patients had not previously received CPIs, whereas in Group 2 (CPI-relapsed), patients had to have achieved stable disease or better on two consecutive scans following PD-1/PD-L1 inhibition (either alone or in combination with chemotherapy) before progressing. Patients were excluded if they had microsatellite instability-high (MSI-high) adenocarcinoma. Further eligibility is listed in the Supplementary Material.

Patients received 200 mg of intravenous (IV) pembrolizumab every three weeks (Q3W) in combination with 3 mg/kg of IV bavituximab weekly until disease progression, discontinuation due to toxicity, withdrawal of consent, study termination, or a maximum of 35 cycles of Q3W treatment. The first 3 patients enrolled were monitored for dose-limiting toxicities (DLTs) (DLT definitions are listed in the Supplementary Material). Following the treatment for at least 1 cycle (3 weeks) of the first 10 eligible patients, safety data from these patients were also reviewed by the safety review committee to confirm the recommended dose for expansion (RDE).

### 2.2. Outcomes

The primary objectives of this study were to assess the safety and tolerability of bavituximab in combination with pembrolizumab in patients with advanced gastric/GOJ adenocarcinoma, and to assess the antitumor activity of the treatment combination based on RECIST version 1.1. Secondary objectives included further characterization of the antitumor activity through additional assessments of clinical benefit, the evaluation of bavituximab pharmacokinetics when administered alongside pembrolizumab, and assessment of the potential immunogenicity of bavituximab.

### 2.3. Exploratory Analysis

We explored whether immune biomarkers, including the neutrophil-to-lymphocyte ratio (NLR), the PD-L1 combined positive score (CPS) and a novel RNA-based gene expression signature (Xerna TME panel), correlated with efficacy outcomes in patients treated with the bavituximab–pembrolizumab combination. The Xerna TME Panel identifies 4 subtypes for the tumour microenvironment using an artificial neural network algorithm of approximately 100 genes to compute a score for two nodes that roughly correspond to “abnormal/pathological blood vessels” (or angiogenesis) and “immune” gene expression. Scores range from low (−1) to high (+1) for each of the angiogenic and immune scores. The four subtypes are characterized as: Angiogenesis (A), Immune Suppressed (IS), Immune Active (IA) and Immune Desert (ID). Data were analysed with the Xerna TME Panel using previously described methods [33,36,37]. To assess associations between TME subtypes and tumour response after bavituximab and pembrolizumab, subtypes associated with a high immune score (IA and IS) were classified as immune-high or biomarker-positive (B+), and subtypes associated with a low immune score (A and ID) were classified as immune-low or biomarker-negative (B−). RNA expression in pre-treatment tumour tissue was analysed using the Xerna TME Panel to prospectively test the hypothesis that tumours with Immune Active (IA) or Immune Suppressed (IS) TME subtypes (B+) are more likely to respond to bavituximab plus pembrolizumab than Angiogenesis (A) or Immune Desert (ID) TME subtypes (B−).

### 2.4. Statistical Analysis

This study had two primary endpoints: (a) the incidence and severity of adverse events (AEs) and serious adverse events (SAEs), graded per NCI CTCAE v5.0, including changes in clinical laboratory parameters; and (b) the investigator-assessed objective response rate (ORR) per RECIST 1.1, defined as the percentage of patients achieving complete or partial responses (CRs and PRs). Secondary endpoints included duration of response (DoR), measured from the first CR or PR until disease progression or death; time to response (TTR), defined as the interval from treatment initiation to the first confirmed CR or PR; disease control rate (DCR), calculated as the percentage of patients achieving CR, PR, or stable disease (SD) at least 6 weeks after the first dose; progression-free survival (PFS), defined as the time from the first dose to disease progression or death; and overall survival (OS), defined as the time from the first dose to death. Additional secondary measures included bavituximab concentrations pre- and post-infusion and the presence of anti-drug antibodies (ADAs). The exploratory analysis evaluated efficacy outcomes (ORR, DCR, PFS, and OS) in relation to predefined biomarker and clinical subgroups (baseline NLR, Xerna TME Panel phenotypes, PD-L1 CPS, MSI status, and prior lines of therapy). Patient demographics were summarized using descriptive statistics. OS, PFS and DoR were estimated using the Kaplan–Meier method with associated 95% confidence intervals (CI). ORRs and DCR were calculated as the percentage of evaluable patients achieving the respective responses, with associated 95% CIs. The exploratory analysis assessed the correlation between tissue and blood biomarkers (baseline NLR, Xerna TME phenotypes, CPS) with efficacy endpoints. The NLR cut-off of 4 was selected as a study-specific exploratory threshold because it was close to the cohort median NLR of 3.3 and consistent with commonly used cut-offs in the literature, while recognizing that it is not an established predictive standard and should therefore be interpreted cautiously.

A sample size of 80 patients across two groups was chosen to ensure minimum enrolment in each of four biomarker genomic-signature groups, enabling exploration of differential treatment responses. For CPI-naive patients, an ORR of less than 15% was considered a threshold for futility as it was unlikely to exceed the single agent activity of pembrolizumab in MMR proficient gastric/GOJ adenocarcinomas (sample size criteria are detailed in the Supplementary Material).

## 3. Results

### 3.1. Patient Characteristics

**Figure 1:**
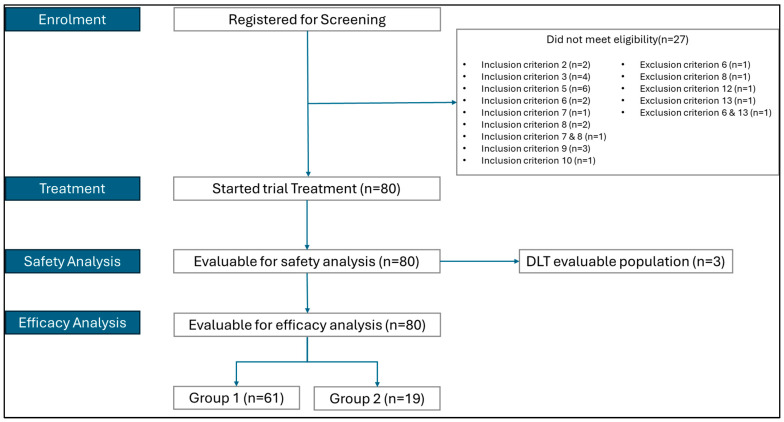
Consort diagram. Flow diagram outlining patient disposition and reasons for exclusion throughout this study. DLT: Dose limiting toxicity. Group 1: Checkpoint inhibitor (CPI) naïve, Group 2: CPI-relapsed.

Between June 2019 and June 2021, a total of 107 patients were screened for this study, of whom 80 received treatment and were evaluated for safety and efficacy: 61 in the CPI-naïve cohort and 19 in the CPI-relapsed cohort (Supplementary Table S1; Figure 1). Baseline characteristics are detailed in Table 1 and prior cancer medications in Supplementary Table S2. The majority of participants were male (75%) and the overall median age was 62.5 years (range: 21–82). All but one patient had an ECOG performance status of 0 or 1. Over 90% of patients had stage IV cancer, and 71.3% had gastric and 28.8% GOJ adenocarcinoma. PD-L1 CPS was <1 in 30% of patients, ≥1 in 61.3%, and ≥10 in 28.8%. The median baseline NLR was 3.3, with 63.8% of patients having an NLR < 4. All patients had undergone prior cancer treatment, including fluoropyrimidines, platinum agents, taxanes, and/or ramucirumab (Table 1). Among these, 46.3% (*n* = 37) received trial treatment as second line therapy, 28.8% (*n* = 23) as third line and 25% (*n* = 20) as fourth or subsequent line therapy.

### 3.2. Efficacy

Median follow-up was 18.7 months (95% CI: 15.4, 20.2) for the CPI-naïve cohort and 9.1 months (95% CI: 5.5, 14.2) for the CPI-relapsed cohort. Table 2 shows the responses in Groups 1 and 2. In the CPI-naïve cohort, among the 61 patients evaluable for the primary efficacy endpoint, the objective response rate (ORR) was 13.1% (95% CI: 5.8, 24.2), with two patients (3.3%) achieving CR and six (9.8%) achieving PR. In the CPI-relapsed cohort, the ORR was 5.3% (95% CI: 0.1, 26.0) among 19 evaluable patients, with no CRs and one patient (5.3%) achieving PR. DCR in the CPI-naïve cohort was 39.3% (95% CI: 27.1–52.7) and 52.6% (95% CI: 28.9, 75.6) in the CPI-relapsed cohort (Table 2). The median TTR for the eight patients in the CPI-naïve cohort who achieved CR or PR was 1.5 months (range: 1 to 5 months) and the median DoR was 12.5 months (95% CI: 2.7–NE) (Supplementary Table S3). In the CPI-relapsed cohort, the single patient who achieved PR had a TTR of 4.1 months and a DoR of 5.8 months. Although not striking at first glance, these outcomes should be interpreted in the context of a heavily pretreated population with limited therapeutic options.

**Figure 2:**
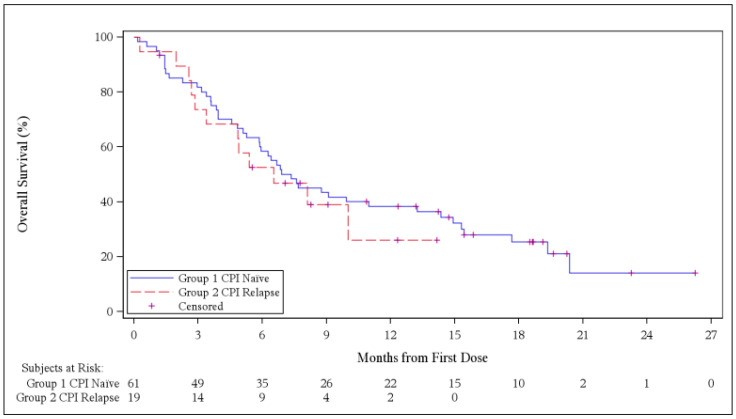
Kaplan–Meier Curve of Overall Survival (Efficacy Population). Overall survival from the first dose in the efficacy population, stratified by prior checkpoint inhibitor (CPI) exposure. CPI-naïve patients (Group 1, solid blue line) and CPI-relapsed patients (Group 2, dashed red line) are shown. Censored observations are indicated by “+”. Numbers at risk are provided below the *x*-axis.

**Figure 3:**
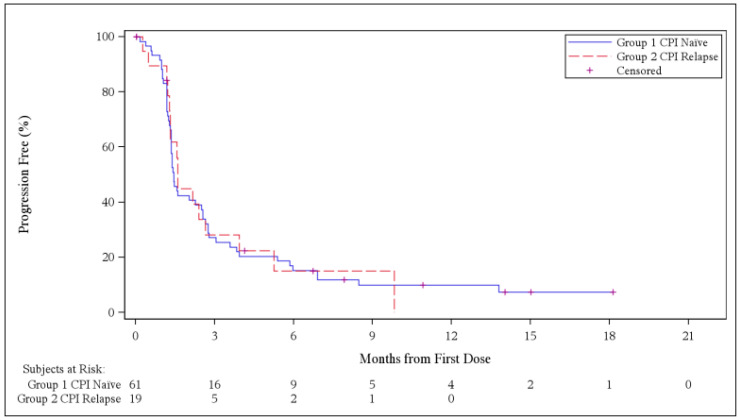
Kaplan–Meier Curve of Progression-Free Survival (Efficacy Population). Progression-free survival from the first dose in the efficacy population, stratified by prior checkpoint inhibitor (CPI) exposure. CPI-naïve patients (Group 1, solid blue line) and CPI-relapsed patients (Group 2, dashed red line) are shown. Censored observations are indicated by “+”. Numbers at risk are provided below the *x*-axis.

The median OS (mOS) was 7.4 months (95% CI: 5.3, 13.2) in the CPI-naïve cohort and 6.5 months (95% CI: 2.9, NE) in the CPI-relapsed cohort (Supplementary Table S4; Figure 2). The twelve-month OS rate was 38.3% and 26.0% for Groups 1 and 2, respectively. The median PFS (mPFS) in the CPI-naïve cohort was 1.4 months (95% CI: 1.3, 2.5), and 1.6 months (95% CI: 1.3, 2.7) in the CPI-relapsed cohort (Supplementary Table S5; Figure 3).

### 3.3. Biomarker-Correlated Outcomes

The distribution of the TME panel phenotypes (Table 1) was consistent across groups, except for a higher prevalence of IS in the CPI-relapsed cohort (42.1%) compared to the CPI-naïve cohort (16.4%). The exploratory biomarker-correlated outcomes for the CPI-naïve cohort are detailed in Supplementary Tables S10 and S11. For the CPI-relapsed cohort (CPI Relapse), meaningful correlation was not feasible as only one patient achieved a partial response. In the CPI-naïve cohort, ORR rates were higher in B+ patients (21.9%; 95% CI: 9.3–40.0), compared to B− patients (4.0%; 95% CI: 0.1–20.4) (Supplementary Table S10). Interestingly, patients with PD-L1 CPS < 1 showed numerically higher ORR (17.6%; 95% CI: 3.8, 43.4) compared to those with CPS ≥ 1 (12.5%; 95% CI: 4.2, 26.8). Similarly, patients with baseline NLR < 4 had a higher ORR (17.9%, 95% CI: 7.5, 33.5) compared to NLR ≥ 4 (4.5%, 95% CI: 0.1, 22.8) (Supplementary Table S11). Additionally, patients with B+/NLR < 4 had an ORR of 33% compared to no responses seen in B+/NLR ≥ 4 (Supplementary Table S11). These trends extended to OS, albeit not to PFS, as patients with NLR < 4 demonstrated longer mOS (11.0 months) compared to those with NLR ≥ 4 (4.7 months).

### 3.4. Safety

The safety analysis included all 80 patients who received treatment. No dose-limiting toxicities (DLTs) were observed, and the safety run-in phase established the recommended bavituximab dose at 3 mg/kg weekly for expansion. Among those experiencing treatment-emergent adverse events (TEAEs), 29 patients (36.3%) experienced at least one Grade 3 TEAE (Supplementary Table S6). Grade 5 TEAEs were reported in 22 patients (27.5%); however, it is important to clarify that deaths were primarily attributed to the underlying cancer (Supplementary Table S7). Notably, none of the serious TEAEs resulting in death were attributed to the study drugs.

The most common TEAEs reported in at least 30% of patients were fatigue (32.5%), constipation (31.3%), and decreased appetite (31.3%) (Table 3). At least one Grade ≥ 3 TEAE was reported by 65% of the patients, most commonly anaemia (12.5%) (Supplementary Table S8). Bavituximab-related TEAEs were reported by 36.3% of patients, with the most frequent being myalgia (8.8%), nausea (7.5%) and headache (6.3%) (Supplementary Table S9). The most frequently reported Grade ≥ 3 bavituximab-related TEAE was anaemia in two patients (3.3%).

Serious (Grade ≥ 3) TEAEs related to the study treatment were documented in five patients (6.3%) (Supplementary Table S6). TEAEs led to the discontinuation of bavituximab and/or pembrolizumab in four patients (6.6%) within the CPI-naïve cohort, but only one patient experienced a study treatment-related TEAE resulting in drug withdrawal (Supplementary Table S6). TEAEs caused treatment reductions or interruptions in 45.9% of the CPI-naïve patients and 26.3% of the CPI-relapsed patients (Supplementary Table S6). No obvious trends were identified for clinical laboratory evaluations in any group. Overall, these findings underscore a manageable safety profile for bavituximab and pembrolizumab in this patient population.

### 3.5. Pharmacokinetic, Pharmacodynamic, and Immunogenicity Analyses

Pharmacokinetic, pharmacodynamic, and immunogenicity analyses were performed on the safety population (N = 80). End-of-infusion (EOI) and trough concentrations of bavituximab were comparable between the CPI-naïve cohort and the CPI-relapsed cohort at all evaluated time points (Supplementary Figures S3 and S4). Similarly, the range of β2-glycoprotein I (β2GP1) concentrations, which bavituximab forms a complex along with phosphatidylserine, was consistent between both groups and across treatment cycles (S5).

At baseline, 66 patients (82.5%) were ADA-negative, 52 patients (85.2%) were ADA-negative in the CPI-naïve cohort and 14 patients (73.7%) were ADA-negative in the CPI-relapsed cohort. During treatment, 27 patients (51.9%) in the CPI-naïve cohort experienced seroconversion, with 16 seroconverting after the first treatment cycle. In the CPI-relapsed cohort, five patients (35.7%) experienced seroconversion, three of whom seroconverted after the first treatment cycle (Supplementary Table S12).

## 4. Discussion

This Phase 2 multinational, open-label, non-randomized study evaluated the safety, tolerability, and anti-tumour activity of bavituximab combined with pembrolizumab in advanced gastric or GOJ adenocarcinoma patients. Neither CPI-naïve nor CPI-relapsed patients experienced DLTs or delayed DLTs, confirming the recommended dose for expansion. The combination of bavituximab and pembrolizumab showed modest overall efficacy and did not meet the pre-specified efficacy threshold. Albeit this should be interpreted in the context of a heavily pretreated population, with most patients having received more than two prior lines of therapy.

Against this background, the limited activity seen in our study is broadly consistent with historical pembrolizumab data in previously treated gastric/GOJ adenocarcinoma. In KEYNOTE-059, pembrolizumab monotherapy in third-line or later disease produced an objective response rate of 11.6% overall and 15.5% in PD-L1-positive tumours, with durable responses in a subset of patients [38]. In KEYNOTE-061, pembrolizumab did not significantly improve overall survival versus paclitaxel in the second-line PD-L1 CPS greater than or equal to one population, and response rates remained modest overall, despite a longer duration of response with pembrolizumab (16.3% vs. 13.6%) [39]. These findings underscore the difficulty of achieving meaningful response rates with PD-1-based therapy in previously treated gastric/GOJ adenocarcinoma, particularly in later-line settings.

At the same time, this study is among the first prospective studies in gastric/GOJ adenocarcinomas to evaluate the combination of a CPI with another novel agent in patients previously treated with prior CPI therapy. The role of immunotherapy following progression on CPI remains a compelling area of research, though data in this setting are still limited. Recently, a Phase 2 study exploring the combination of pembrolizumab with regorafenib in patients with advanced hepatocellular carcinoma (HCC) who have previously progressed on CPI, reported similarly modest activity [40]. Evidence for such combinations in other malignancies is also sparse. However, promising initial outcomes were observed in the GDFATHER-1/2a trial, which demonstrated durable responses in select patients with non-squamous non-small cell lung cancer and urothelial cancer who were refractory to prior CPI treatment when treated with the neutralizing GDF-15 antibody, visugromab, in combination with nivolumab [41].

The most common TEAEs of any grade reported in more than one third of patients included gastrointestinal disorders and fatigue, whereas anaemia was the most common grade ≥3 TEAE. The safety profile indicates that the treatment combination of bavituximab plus pembrolizumab was generally safe and well tolerated with no new safety signals identified. The overall toxicity profile was consistent with previous trials of bavituximab and aligned with expectations for pembrolizumab monotherapy, with no additional toxicities observed [30,33].

The DCR in our study was encouraging, ranging from 39.3% to 52.6%, and responses were durable, with a median DoR reaching 12.5 months in the CPI-naïve patients. Notably, efficacy was more pronounced in patients with B+ status, suggesting a potential prognostic, or even predictive role for this TME signature. Similarly, CPI-naïve patients classified as ID exhibited the longest mOS. Additionally, NLR emerged as a prognostic biomarker, consistently associated with improved outcomes, showing higher ORRs and OS. Interestingly, combining NLR with the TME biomarker signature identified a patient population most likely to benefit from bavituximab plus pembrolizumab, with patients having B+/NLR < 4 achieving the highest ORR in this study.

Although the combination of bavituximab and pembrolizumab did not show significant benefit in this study, the SUNRISE Phase 3 trial in lung cancer indicated that prior treatment with bavituximab improved OS in patients who later received CPI [30]. This suggests that PS inhibition may enhance the efficacy of immunotherapy, as supported by the immunomodulatory effects observed in preclinical models [15,20,28]. In HCC the combination of bavituximab with pembrolizumab met its primary endpoint in a Phase 2 study, with many responses being durable [33]. In our study, the duration of response was 12 months for the CPI-naïve group, with most responses observed in the B+ subgroup, which is expected to be more responsive to CPIs. The combination demonstrated similar or better ORR in patients with CPS < 1, perhaps suggesting it could remain effective even in PD-L1-negative populations. Additionally, the NLR status emerged as a significant distinguishing factor with this combination, particularly given the relative lack of biomarkers, with PD-L1 CPS score, MSS/MSI status and MMR status being the only validated biomarkers for pembrolizumab so far. Furthermore, OS and PFS in the CPI-relapse group were similar to the CPI-naïve group, suggesting the potential role of resistance circumvention to CPI with bavituximab.

The lack of a control arm limits the attribution of outcomes to the combination therapy, particularly given modest efficacy signals. Although potential predictors of treatment response were identified in this study, results should be viewed as preliminary due to the small sample size assessed, the lack of randomization or blinding and the presence of potential confounders. Likewise, evaluating the predictive effect of biomarker status in CPI-relapsed patients was not feasible; the CPI-relapsed cohort was small, with only a single responder, which limits meaningful interpretation. The biomarker findings involving the TME panel and the neutrophil-to-lymphocyte ratio are intriguing. These remain exploratory and require validation in larger, controlled studies. The lack of multivariate analysis in our study further reduces their predictive value.

## 5. Conclusions

Overall, despite bavituximab plus pembrolizumab being well tolerated, efficacy in this pre-treated population was not very encouraging. However, the results highlight the potential of a PS-targeting agent in biomarker-selected populations, warranting further studies to explore this pathway. With CPIs increasingly used in earlier treatment settings, novel immunotherapy combinations with refined biomarker-based selection will be especially important for patients with prior CPI exposure. Additionally, the impact of PS on the TME and immunomodulatory role, coupled with its selective expression on tumours and the associated vascular endothelium, underscores its potential as a target for novel therapies such as antibody–drug conjugates, bispecific antibodies, CAR-T cells, or as a chemoattractant. These findings emphasize the need for further innovation to broaden the benefits of immunotherapy for patients with gastric/GOJ adenocarcinomas.

## Figures and Tables

**Table 1:** Baseline patient characteristics.

	Group 1 CPI Naïve *n* = 61	Group 2 CPI Relapse *n* = 19	Overall *n* = 80
**Sex**, *n* (%)			
Male	45 (73.8%)	15 (78.9%)	60 (75.0%)
Female	16 (26.2%)	4 (21.1%)	20 (25.0%)
**Age at Screening**			
Mean (SD)	60.2 (12.82)	61.2 (11.68)	60.4 (12.49)
Median	62.0	65.0	62.5
Range	21–82	35–81	21–82
**Race**, *n* (%)			
White	21 (34.4%)	4 (21.1%)	25 (31.3%)
African American	3 (4.9%)	0	3 (3.8%)
Asian	33 (54.1%)	12 (63.2%)	45 (56.3%)
Other	4 (6.6%)	3 (15.8%)	7 (8.8%)
**Baseline ECOG Performance Status**, *n* (%)			
0	19 (31.1)	3 (15.8)	22 (27.5)
1	41 (67.2)	16 (84.2)	57 (71.3)
2	1 (1.6)	0	1 (1.3)
**Site of Disease**, *n* (%)			
Gastric	43 (70.5%)	14 (73.7%)	57 (71.3%)
GOJ	18 (29.5)	5 (26.3%)	23 (28.8%)
**TNM Staging of Diagnosis at Study Entry**, *n* (%)			
IIIb	2 (3.3%)	1 (5.3%)	3 (3.8%)
IV	59 (96.7%)	18 (94.7%)	77 (96.3%)
**Siewert Classification**, *n* (%)			
Type 1	15 (24.6%)	4 (21.1%)	19 (23.8%)
Type 2	4 (6.6%)	0	4 (5.0%)
Type 3	11 (18.0%)	1 (5.3%)	12 (15.0%)
Not Available	31 (50.8%)	14 (73.7%)	45 (56.3%)
**HER2 Status at Diagnosis**, *n* (%)			
Positive	10 (16.4%)	3 (15.8%)	13 (16.3%)
Negative	51 (83.6%)	15 (78.9%)	66 (82.5%)
Unknown	0	1 (5.3%)	1 (1.3%)
**Epstein–Barr virus**, *n* (%)			
Positive	1 (1.6%)	3 (15.8%)	4 (5.0%)
Negative	10 (16.4%)	6 (31.6%)	16 (20.0%)
Unknown	50 (82.0%)	10 (52.6%)	60 (75.0%)
**MSI Status at Screening**, *n* (%)			
MSI High	0	1 * (5.3%)	1 * (1.3%)
MSS (Non-MSI)	43 (70.5%)	16 (84.2%)	59 (73.8%)
Unknown	18 (29.5%)	2 (10.5%)	20 (25.0%)
**Central PD-L1 CPS**, *n* (%)			
<1	17 (27.9%)	7 (36.8%)	24 (30.0%)
≥1	40 (65.6%)	9 (47.4%)	49 (61.3%)
≥1 and <10	22 (36.1%)	4 (21.1%)	26 (32.5%)
≥10	18 (29.5%)	5 (26.3%)	23 (28.8%)
Missing	4 (6.6%)	3 (15.8%)	7 (8.8%)
**TME Panel**, *n* (%)			
IA	22 (36.1%)	6 (31.6%)	28 (35.0%)
ID	14 (23.0%)	4 (21.1%)	18 (22.5%)
A	11 (18.0%)	1 (5.3%)	12 (15.0%)
IS	10 (16.4%)	8 (42.1%)	18 (11.5%)
Missing	4 (6.6%)	0	4 (5.0%)
**Biomarker Status**, *n* (%)			
B+	32 (52.5%)	14 (73.7%)	46 (57.5%)
B−	25 (41.0%)	5 (26.3%)	30 (37.5%)
Missing	4 (6.6%)	0	4 (5.0%)
**Baseline NLR**			
Mean (SD)	4.1 (2.95)	4.7 (3.01)	4.3 (2.96)
Median	3.2	3.5	3.3
Minimum, Maximum	1, 18	1, 10	1, 18
**Baseline NLR**, *n* (%)			
<4	39 (63.9%)	12 (63.2%)	51 (63.8%)
≥4	22 (36.1%)	7 (36.8%)	29 (36.3%)
**Prior Radiotherapy**, *n* (%)			
Yes	13 (21.3%)	8 (42.1%)	21 (26.3%)
No	48 (78.7%)	11 (57.9%)	59 (73.8%)
**Prior Cancer Surgery**, *n* (%)			
Yes	37 (60.7%)	9 (47.4%)	46 (57.5%)
No	24 (39.3%)	10 (52.6%)	34 (42.5%)
**Previous Lines of Therapy**, *n* (%)			
1	35 (57.4%)	2 (10.5%)	37 (46.3%)
2	16 (26.2%)	7 (36.8%)	23 (28.8%)
3	8 (13.1%)	8 (42.1%)	16 (20.0%)
4 or More	2 (3.3%)	2 (10.5%)	4 (5.0%)
**Cancer Medication Indication**, *n* (%)			
Neo-Adjuvant	5 (8.2%)	2 (10.5%)	7 (8.8%)
Adjuvant	6 (9.8%)	2 (10.5%)	8 (10.0%)
Palliative	50 (82.0%)	14 (73.7%)	64 (80.0%)
Other	0	1 (5.3%)	1 (1.3%)
**Best Response on Latest Prior Therapy**, *n* (%)			
Complete Response	2 (3.3%)	1 (5.3%)	3 (3.8%)
Partial Response	8 (13.1%)	9 (47.4%)	17 (21.3%)
Stable Disease	30 (49.2%)	9 (47.4%)	39 (48.8%)
Progressive Disease	15 (24.6%)	0	15 (18.8%)
Unknown	6 (9.8%)	0	6 (7.5%)
**Reason for Latest Prior Therapy Discontinuation**, *n* (%)			
Treatment Completed	2 (3.3%)	1 (5.3%)	3 (3.8%)
Progressive Disease	55 (90.2%)	18 (94.7%)	73 (91.3%)
Intolerance	3 (4.9%)	0	3 (3.8%)
Other	1 (1.6%)	0	1 (1.3%)

Abbreviations: ECOG: Eastern Cooperative Oncology Group; CPI: Immune checkpoint inhibitor; CPS: combined positive score; HER2: Human epidermal growth factor receptor 2; MSI: microsatellite instability; MSS: microsatellite stable; A: Angiogenic; IA: Immune active; ID: Immune desert; IS: immune suppressed; NLR: Neutrophils/lymphocyte ratio; PD-L1: Programmed death-ligand 1; TNM: Tumour, Node, Metastasis; GOJ: Gastroesophageal junction; SD: standard deviation. * Protocol deviation.

**Table 2:** Summary of Best Response and Overall Response to Treatment (RECIST v1.1) (Efficacy Population).

Parameter	Group 1 (CPI Naïve, *n* = 61)	Group 2 (CPI Relapse, *n* = 19)
**Best Response**, *n* (%)		
CR	2 (3.3%)	0
PR	6 (9.8%)	1 (5.3%)
SD	16 (26.2%)	9 (47.4%)
PR, Unconfirmed	2 (3.3%)	0
PD	31 (50.8%)	8 (42.1%)
NE	6 (9.8%)	1 (5.3%)
**Overall Response Rate (CR or PR) {1}**	8 (13.1%)	1 (5.3%)
95% CI {2}	(5.8, 24.2)	(0.1, 26.0)
**Disease Control Rate (CR or PR or SD) {3}**	24 (39.3%)	10 (52.6%)
95% CI {2}	(27.1, 52.7%)	(28.9, 75.6%)

Abbreviations: CI: confidence interval; CPI: immune check point inhibitor therapy; CR: complete response; NE: not evaluable; PD: progressive disease; PR: partial response; SD: stable disease. {1} The Overall Response Rate was defined as the proportion of patients with a confirmed best response of CR or PR. {2} The two-sided 95% CI was calculated using the Clopper–Pearson method. {3} The DCR was defined as the proportion of patients with Overall Response or with any assessment of SD, PR or CR evaluated at 6 weeks (not less than 35 days) after the first dose of study treatment.

**Table 3:** Most Common Treatment-Emergent Adverse Events of any Grade (Safety Population).

SOC Preferred Term	Group 1 CPI Naïve *n* = 61	Group 2 CPI Relapse *n* = 19	Overall *n* = 80
	N (%)		
Patients with at least one TEAE	61 (100%)	19 (100%)	80 (100%)
**Blood and lymphatic system disorders**	14 (23.0%)	3 (15.8%)	17 (21.3%)
Anaemia	14 (23.0%)	3 (15.8%)	17 (21.3%)
**Gastrointestinal disorders**	53 (86.9%)	16 (84.2%)	69 (86.3%)
Constipation	16 (26.2%)	9 (47.4%)	25 (31.3%)
Nausea	18 (29.5%)	5 (26.3%)	23 (28.8%)
Diarrhoea	16 (26.2%)	5 (26.3%)	21 (26.3%)
Vomiting	15 (24.6%)	3 (15.8%)	18 (22.5%)
Abdominal pain	13 (21.3%)	4 (21.1%)	17 (21.3%)
**General disorders and administration site conditions**	39 (63.9%)	10 (52.6%)	49 (61.3%)
Fatigue	20 (32.8%)	6 (31.6%)	26 (32.5%)
**Metabolism and nutrition disorders**	33 (54.1%)	6 (31.6%)	39 (48.8%)
Decreased appetite	21 (34.4%)	4 (21.1%)	25 (31.3%)
**Musculoskeletal and connective tissue disorders**	30 (49.2%)	8 (42.1%)	38 (47.5%)
Myalgia	7 (11.5%)	5 (26.3%)	12 (15.0%)
**Nervous system disorders**	20 (32.8%)	4 (21.1%)	24 (30.0%)
Headache	7 (11.5%)	4 (21.1%)	11 (13.8%)
**Respiratory, thoracic and mediastinal disorders**	23 (37.7%)	8 (42.1%)	31 (38.8%)
Dyspnoea	11 (18.0%)	5 (26.3%)	16 (20.0%)

Abbreviations: CPI = immune check point inhibitor therapy; SOC = system organ class; TEAE = treatment-emergent adverse event. Notes: Within a SOC, patients may have reported more than one preferred term. Patients are counted once for each preferred term and each SOC.

## Data Availability

The original contributions presented in this study are included in the article/Supplementary Material. Further inquiries can be directed to the corresponding author.

## Supplementary Materials

The following supporting information can be downloaded at: https://www.mdpi.com/article/10.3390/curroncol33060319/s1.

## Abbreviations

The following abbreviations are used in this manuscript:

ADAs	Anti-drug antibodies
ADCC	Antibody-dependent cellular cytotoxicity
AEs	Adverse events
B+	Biomarker-positive
B−	Biomarker-negative
CPI	Immune checkpoint inhibitor
CPS	Combined positive score
CR	Complete response
DCR	Disease control rate
DLT	Dose-limiting toxicity
DoR	Duration of response
EOI	End-of-infusion
GEAs	Gastroesophageal adenocarcinomas
GOJ	Gastroesophageal junction
HCC	Hepatocellular carcinoma
IA	Immune Active
ID	Immune Desert
IgG1	G1 immunoglobulin
IS	Immune Suppressed
IV	Intravenous
MMR	Mismatch repair
MSI-high	Microsatellite instability—high
MSS	Microsatellite—stable
NLR	Neutrophil-to-lymphocyte ratio
ORR	Objective response rate
OS	Overall survival
PFS	Progression-free survival
PR	Partial response
PS	Phosphatidylserine
Q3W	Every three weeks
RDE	Recommended dose for expansion
SAEs	Serious adverse events
SD	Stable disease
TEAEs	Treatment-emergent adverse events
TME	Tumour microenvironment
TTR	Time to response
