# Supplementary material for: Targeting Phosphatidylserine in Advanced Gastric and Gastroesophageal Junction Adenocarcinomas: A Phase 2 Trial of Bavituximab Plus Pembrolizumab with Biomarker-Correlated Outcomes
Source: Curr Oncol. 2026 May 28;33(6):319. doi: 10.3390/curroncol33060319 (PMC13298944; doi:10.3390/curroncol33060319)
Supplement: Supplementary file 1 [file curroncol-33-00319-s001.zip › curroncol-4269769-supplementary.pdf]

Supplementary Material

Contents

Supplementary Material 1

1. Methods 3

1.1 Patient Inclusion Criteria 3

1.2 Patient Exclusion Criteria 6

1.3 Procedures 8

1.4 Definition of Dose-limiting Toxicities 8

1.5 Outcomes 9

1.6 Efficacy Outcomes Definitions 10

1.7 Determination of Sample Size 11

2. Results 13

2.1 Study Population 13

- Supplementary Table 1. Patient Disposition 13
- Supplementary Table 2x. Prior Cancer Medications 14

2.2 Efficacy Outcomes 14

- Supplementary Table 2. Summary of time to Response and duration of Response – Group 1 (CPI Naïve) (Efficacy population) 15
- Supplementary table 3. Summary of Overall Survival (Efficacy population) 16
- Supplementary Table 4. Summary of Progression-Survival (Efficacy population) 16

2.3. Safety 17

- Supplementary Table 5. Overall Summary of Treatment-Emergent Adverse Events (Safety Population) 17
- Supplementary Table 6. Serious TEAEs Leading to Death by SOC and Preferred Term (Safety Population) 18
- Supplementary Table 7. TEAEs Severity Grade ≥ 3 by Preferred Term in ≥ 10% Patients in Any Group (Safety Population) 19
- Supplementary Table 8. Treatment-related TEAEs by Preferred Term in ≥ 10% Patients in Any Group (Safety Population) 19

2.3 Biomarker-driven Outcomes 20

- Supplementary Table 9: Summary of TME Panel-1 and Biomarker Status (Safety Population) .... **Error! Bookmark not defined.**
- Supplementary Table 10: Key Efficacy Parameters by TME Panel-1 Scores and Biomarker Status (Efficacy Population) - Group 1 CPI Naïve 20

- Supplementary Table 11: Key Efficacy Parameters by Subgroups (Efficacy Population) - Group 1 CPI Naïve 21
- Supplementary Figure 1. Individual Neutrophils/Lymphocytes (NLR) Percent Change from Baseline by Treatment Cycle in Group 1 (CPI-naïve, Safety Population) 22
- Supplementary Figure 2. Individual Neutrophils/Lymphocytes (NLR) Percent Change from Baseline by Treatment Cycle in Group 2 (CPI-relapse, Safety Population) 22

2.4 Pharmacokinetic, pharmacodynamic, and immunogenicity analyses 24

- Supplementary Figure 3: Individual Bavituximab Pharmacokinetic Concentrations Versus Treatment Cycle in Group 1 (CPI-naïve, Safety Population) 24
- Supplementary Figure 4: Individual Bavituximab Pharmacokinetic Concentrations Versus Treatment Cycle Safety Population in Group 2 (CPI-relapse, Safety Population) 25
- Supplementary table 12: Summary of Anti-Drug Antibodies (ADA) (Safety Population) 25
- Supplementary Figure 5: ß2-GP1 Mean Change from Baseline Concentrations Versus Treatment Cycle (Safety Population) 26

1. Methods

1.1 Patient Inclusion Criteria

Patients must meet all the following inclusion criteria to be enrolled.

For Group 1 only (CPI naïve):

1. Progressed on and/or after at least 1 prior regimen for metastatic disease which includes a fluoropyrimidine and a platinum therapy:

• Progression within 6 months of prior adjuvant or neoadjuvant chemotherapy will be deemed a rapid progressor and thus, equivalent to 1 advanced/metastatic disease treatment regimen.

• Changing from IV to oral fluoropyrimidine without noted progression is considered only 1 prior regimen.

• Human epidermal growth factor receptor 2 (HER2)-positive patients must have received prior anti-HER2 therapy and demonstrate PD or was ineligible for such therapy.

For Group 2 only (CPI relapse):

2. Patient achieved stable disease or better in two consecutive scans to PD-1/PD-L1 inhibition alone or in combination with chemotherapy and relapsed following PD-1/PD-L1 inhibition either alone or in combination with chemotherapy

• All patients must be immediate (defined by within 3 months) progressors of PD-1/PD-L1 inhibition with no intervening treatment with other agents such as chemotherapy alone.

For both Group 1 and Group 2:

3. Signed written informed consent obtained prior to performing any study procedure, including screening procedures.

4. Men and women ≥ 18 years old; ≥ 20 years old in South Korea and Taiwan.

5. Pathologically documented unresectable metastatic or locally advanced gastric or GEJ adenocarcinoma

• Must be metastatic/unresectable at the time of enrolment into this study

6. Willing and able to provide fresh (since most recent progression) formalin-fixed paraffin-embedded tissue tumour sample for screening of signature status prior to study treatment and to measure PD-1 status. An archival tissue sample should also be provided, if available.

7. Presence of at least one measurable lesion assessed by the Investigator per RECIST version 1.1.

8. Eastern Cooperative Oncology Group performance status (ECOG PS) of 0 or 1.

9. Has adequate organ functions defined as:

System	Laboratory Value
Hematological	
Absolute neutrophil count (ANC)	≥ 1.5 × 10^9/L
Platelets	≥ 100 × 10^9/L
Hemoglobin	≥ 9.0 g/dL
Renal	
Dipstick or routine urinalysis	Urine protein < 2 g/24 h
- For proteinuria ≥ 2+ or urine protein/creatinine ratio ≥ 0.5, 24-hour urine must be collected	
Creatinine, or	≤ 1.5 × upper limit of normal (ULN)
Glomerular filtration rate (GFR)	≥ 50 mL/min
Hepatic	
Total bilirubin	≤ 1.5 × ULN (except for known Gilbert's syndrome)
Aspartate transaminase (AST) / alanine transaminase (ALT)	≤ 2.5 × ULN for patients without liver metastases
	≤ 5 × ULN for patients with liver metastases
Coagulation	
International normalized ratio (INR) or prothrombin time (PT)	INR ≤ 1.5 × ULN or PT ≤ 5 sec above ULN, unless the patient is receiving anticoagulant

	therapy, as long as INR or PT is within the therapeutic range of intended use of anticoagulants
Partial thromboplastin time (PTT) or activated partial thromboplastin time (aPTT)	PTT or aPTT ≤ 5 seconds above ULN, unless the patient is receiving anticoagulant therapy, as long as PTT or aPTT is within the therapeutic range of intended use of anticoagulants
Notes:<br>(a) All labs will be performed, and values calculated per local institution standards.<br>(b) Transfusion and/or erythropoietin therapy to increase hemoglobin level is not permitted within 1 week before the baseline hematology profile.<br>(c) Patients on full-dose anticoagulation must be on stable oral anticoagulation or low-molecular-weight heparin for ≥ 14 days. If receiving oral anticoagulants, the patient must have an INR ≤ 3.0, no active bleeding (i.e., no bleeding within 14 days prior to first dose of study therapy), and no pathological condition with a high risk of bleeding (e.g., tumor involving major vessels or known varices).	

10. Women of childbearing potential must have a negative serum or urine pregnancy test within 72 hours prior to start of study treatment.

11. Women must not be breastfeeding.

12. Women of childbearing potential defined as not surgically sterile or have not been free from menses for ≥ 2 years, must agree to follow instructions for highly effective method(s) of contraception as described in Section 4.4 Patients or Partners of Patients of Reproductive Potential for the duration of treatment with study drug bavituximab and pembrolizumab plus 5 months post-treatment completion.

Males who are sexually active with women of childbearing potential must agree to follow instructions for highly effective method(s) of contraception as described in Section 4.4 s or Partners of Patients of Reproductive Potential for the duration of treatment with study treatment plus 90 days post-treatment completion.

13. Has adequate treatment washout period before start of study treatment, defined as:

• Major surgery ≥ 4 weeks; radiation therapy with abdominal radiation ≥ 4 weeks and have recovered from all radiation-related toxicities, not require corticosteroids and not have had radiation pneumonitis; palliative radiation without abdominal radiation ≥ 2 weeks; chemotherapy ≥ 3 weeks; biologic therapy ≥ 3 weeks.

1.2 Patient Exclusion Criteria

Patients who meet any of the following criteria may not be enrolled.

For Group 1 only:

1. Prior treatment with any checkpoint inhibitor or other therapies targeting T-cell control.

For Group 2 only:

2. Primary refractory patients, defined as disease progression at first scan following initiation of PD-1/PD-L1 inhibitor treatment, or if best overall response to PD-1/PD-L1 inhibition was disease progression.

For both Group 1 and Group 2:

3. Received any form of anti-phosphatidylserine therapies.
4. Known MSI-H gastric or GEJ adenocarcinoma
5. Medical history of myocardial infarction within 6 months before registration, symptomatic congestive heart failure (CHF) (New York Heart Association Class II to IV; Appendix C), troponin levels consistent with myocardial infarction as defined according to American College of Cardiologists (ACC) guidelines, unstable angina, or serious cardiac arrhythmia
requiring treatment.
6. Experienced weight loss >10% over 2 months prior to first dose of study treatment.
7. History of (non-infectious) pneumonitis that required steroids or has current pneumonitis.
8. Known active CNS metastases and/or carcinomatous meningitis. Subjects with previously treated brain metastases may participate provided they are stable without evidence of progression via imaging for at least four weeks prior to first dose of study, and no evidence of neurological symptoms. Carcinomatous meningitis is excluded regardless of clinical stability.
9. Known additional malignancy that is progressing or has required active treatment in within the past 3 years.
Note: Participants with basal cell carcinoma of the skin, squamous cell carcinoma of the skin, or carcinoma in situ (e.g., breast carcinoma, cervical cancer in situ) that have undergone potentially curative therapy are not excluded.
10. Active infection requiring systemic therapy.
11. Known human immunodeficiency virus (HIV) infection, or known acute hepatitis B or C infection.
12. Unresolved toxicities from previous cancer treatments (other than alopecia) not yet resolved

to Grade ≤ 1 or baseline. Grade 2 toxicities may be eligible at the discretion of the Investigator after consultation with the Sponsor’s medical monitor.

13. History or current evidence of any condition, therapy, or laboratory abnormality that might confound the results of the study, interfere with the participant's participation for the full duration of the study, or is not in the best interest of the participant to participate, in the opinion of the treating investigator.

14. Active autoimmune disease or history of chronic recurrent autoimmune disease, requiring systemic treatment for the past two years (i.e., disease modifying agents, corticosteroids, or immunosuppressive drugs).

• Replacement therapy (thyroxine, insulin, or physiological corticosteroid replacement for either adrenal or pituitary insufficiency) is not considered a form of systemic treatment.

15. History of hypersensitivity to pembrolizumab and/or any of its excipients that in the opinion of the investigator suggests a high risk for a severe hypersensitivity reaction while on treatment.

16. History of infusion reactions to any component/excipient of bavituximab.

17. History of a hypersensitivity to mAbs that in the opinion of the investigator suggests a high risk for a severe hypersensitivity reaction while on treatment.

18. Systemic glucocorticoid therapy (>10 mg daily prednisone or equivalent) or any other form of immunosuppressive therapy within 7 days prior to the first dose of study treatment (note: topical, inhaled, nasal and ophthalmic steroids are permitted).

19. Received a live vaccine within 30 days prior to the first dose of study drug. Examples of live vaccines include, but are not limited to, the following: measles, mumps, rubella, varicella/zoster (chicken pox), yellow fever, rabies, Bacillus Calmette–Guérin (BCG), and typhoid vaccine. Seasonal influenza vaccines for injection are generally killed virus vaccines and are allowed; however, intranasal influenza vaccines (e.g., FluMist®) are live attenuated vaccines and are not allowed.

20. Prior organ transplantation including allogeneic or autologous stem-cell transplantation.

21. Currently participating in or has participated in a study of an investigational agent or has used an investigational device within 4 weeks prior to the first dose of study treatment.nNote: Participants who have entered the follow-up phase of an investigational study may participate as long as it has been 4 weeks after the last dose of the previous investigational agent.

22. Receipt of treatment with immunotherapy, biological therapies, or therapeutic doses of hormonal therapies within 3 weeks of scheduled C1D1 dosing.

23. Known psychiatric, substance abuse disorder, or geographical travel limitations that would interfere participant’s ability to cooperate with the requirements of the study.

24. Pregnant or breastfeeding or expecting to conceive or father children within the projected duration of the study, starting with the screening visit through 5 months after the last dose of study treatment.

1.3 Procedures

Following the safety run-in of a minimum of three and a maximum of ten patients receiving bavituximab and pembrolizumab, additional patients are to be recruited if the initial dosing was deemed tolerable. If the initial doses of bavituximab were not well tolerated, lower doses of bavituximab could be explored. Once RDE was established from the DLT observation period, dose reductions for bavituximab were not permitted. No dose reductions were allowed for pembrolizumab. Dose interruptions were permitted to manage toxicities, with bavituximab allowed to be paused for up to 21 days. If bavituximab was withheld for more than 21 days, it was permanently discontinued. Pembrolizumab could be suspended for up to 12 weeks. Approximately 80 patients were expected to be enrolled in the study, with a minimum of 40 patients in Group 1 and a minimum of 20 patients in Group 2.

1.4 Definition of Dose-limiting Toxicities

A DLT was defined as any of the AEs described below that occurred during the DLT observation period and was at least possibly related to bavituximab or study treatment. The Investigator used CTCAE v5.0 guidelines to assign an AE term and severity grade relevant to the DLT.

Hematological toxicities as follows:

  - Grade 4 hematologic toxicity lasting ≥7 days, except thrombocytopenia:
  - Grade 4 thrombocytopenia of any duration
  - Grade 3 thrombocytopenia associated with clinically significant bleeding
- ≥ Grade 3 febrile neutropenia
  - Grade 3 was defined as absolute neutrophil count (ANC) 38.3° C (101° F) or a sustained temperature of ≥38o C (100.4° F) for more than 1 hour.
  - Grade 4 was defined as ANC 38.3° C (101° F) or a sustained temperature of ≥38° C (100.4° F) for more than 1 hour, with life-threatening consequences and urgent intervention indicated.
- Grade 4 lymphocyte decreases lasting >14 days

Non-hematologic toxicities:

- Any non-hematologic AE ≥Grade 3 in severity was to be considered a DLT, with the following exceptions: Grade 3 fatigue lasting ≤3 days; Grade 3 diarrhea, nausea, or vomiting without use of anti-emetics or anti-diarrheals per standard of care; Grade 3 rash without use of corticosteroids or anti-inflammatory agents per standard of care.
- Any Grade 3 or Grade 4 non-hematologic laboratory value if:
    - Clinically significant medical intervention was required to treat the patient, or
    - The abnormality led to hospitalization, or the abnormality persisted for >1 week; the abnormality resulted in a Drug-induced Liver Injury
    - Exceptions: Clinically nonsignificant, treatable, or reversible laboratory abnormalities including liver function tests, uric acid, etc.
- Prolonged delay (>2 weeks) in initiating Cycle 2 due to treatment-related toxicity.
- Any treatment-related toxicity that caused the participant to discontinue treatment during Cycle 1.
- Missing >25% of planned doses as a result of drug-related AE(s) during the first cycle.
- Grade 5 toxicity

1.5 Outcomes

Anti-tumour activity was assessed radiographically by the investigator using objective response criteria per RECIST 1.1. Tumour response was assessed 6 weeks after treatment initiation and then every 6 weeks. For patients with progressive disease (PD), a confirmatory scan was optional at least four weeks later to confirm PD. Patients were monitored for all AEs from the time the first dose of study treatment through 30 days after the last dose of study treatment. Adverse events were classified using the Common Terminology Criteria for Adverse Events Version 5.0 (CTCAE V5.0). For irAEs or adverse events of special interest (AESIs) and SAEs, the follow-up period will be 90 days from the last dose of study treatment, or 30 days following cessation of study treatment if the participant initiates new anti-cancer treatment.

The safety population included all patients who received at least 1 dose of bavituximab or pembrolizumab. The DLT-evaluable population included the first 3 patients up to a maximum of 10 patients who (1) receive at least 70% of the dose of bavituximab (2) complete the DLT observation period or discontinue (from study treatment or from the study) during the DLT observation period (Cycle 1) because of a DLT. The PK population included all treated patients who received at least 1 full dose of bavituximab and pembrolizumab and have baseline and at least one postbaseline evaluable

PK sample. The efficacy population included all patients (including DLT patients) who received any quantity of study treatment at the dose recommended for the Phase 2 population.

1.6 Efficacy Outcomes Definitions

ORR

Based on RECIST version 1.1 (Protocol Appendix A) criteria, a patient may achieve as BOR either CR, partial response (PR), SD, or PD. The primary efficacy endpoint was ORR, defined as the percentage of patients achieving a complete response (CR) or partial response (PR) based on RECIST version 1.1. Tumour imaging was performed every 6 weeks (±7 days) after treatment initiation or more frequently if clinically indicated, until one of the following occurred: disease progression, start of new anticancer treatment, withdrawal of consent, death, or end of study. The primary efficacy endpoint, ORR, was calculated as the proportion of patients achieving a complete response (CR) or partial response (PR) among the efficacy population. The ORR and 95% CI were presented for the efficacy population Group 1, Group 2, and overall.

Disease Control Rate (DCR)

Disease control rate (DCR) was defined as the percent of patients in the efficacy population with an objective response (ie, CR or PR) and SD evaluated at 6 weeks or later after the date of first dose. The same analysis approach used for the primary efficacy analysis was applied on DCR.

Time to Response (TTR) & Time to Response

Time to response and DoR were calculated only for patients with a CR or PR response. Duration of response, defined as the interval in months from the date measurement criteria were met for CR or PR (whichever was first recorded) until the earliest date of disease progression, as determined by Investigator assessment of objective radiographic disease assessments per RECIST version 1.1 or death due to any cause if occurring sooner than progression. Duration of response for Group 1 and Group 2 was analyzed using the Kaplan-Meier (KM) method, with associated 95% CI and illustrated graphically using KM curves. Time to response was the interval in months from first treatment until the first date of a confirmed CR or PR response.

OS

Overall survival was defined as the interval in months from the date of first dose of investigational agent until the date of death in safety population. All deaths were included in the analysis. Overall

survival for Group 1 and Group 2 was analysed using the KM method, with associated 95% CI and illustrated graphically using KM curves. Follow-up time for OS analysis was assessed using reverse KM method, reversing censor and event flags. Patients with no known date of death had their survival event time censored at the latest date of last completed visit or patient contact or last known date alive recorded on the eCRF.

PFS

Progression-free survival was defined as the interval in months from the date of first dose of investigational agent until the earliest date of disease progression. Disease progression was determined by Investigator assessment of objective radiographic disease assessments per RECIST version 1.1 or death due to any cause if occurring sooner than progression in efficacy population. Progression-free survival for Group 1 and Group 2 was analysed using the KM method, with associated 95% CI and illustrated graphically using KM curves.

Time to Progression Censoring Rules

Patients lacking evidence of disease progression, including those who initiated non-study anticancer therapy prior to disease progression, had their DoR and PFS event times censored on the last tumour assessment date at which disease status was determined.

1.7 Determination of Sample Size

The sample size of approximately 80 patients across both Group 1 and Group 2 was chosen to allow minimal enrolment within each of our 4 putative biomarker subtypes to allow for exploration of differential responses. An ORR of <15% for Group 1 was regarded as futile as it was unlikely to exceed the activity of pembrolizumab as a single agent in this study population.

The following sample sizes were applied:

- DLT Observation: up to 10 DLT-evaluable patients in total
- Phase 2: Minimum efficacy analysis: 40 total patients across both Group 1 and Group 2; Full enrolment: approximately 80 patients

For the CPI-naïve patient population assumptions, the historical ORR of pembrolizumab in the 2nd-line setting was approximately 15% and an ORR of the bavituximab-pembrolizumab combination was set to 30%. Approximately 40 patients were needed to show a statistical superiority of bavituximab-pembrolizumab with 80% statistical power at one-sided type I error rate of $\alpha = 0.10$. In

the 3rd-line setting if the historical ORR was assumed as 6%, if the true ORR of bavituximab-pembrolizumab combination was 15%, then approximately 40 patients were needed to show a statistical superiority of bavituximab-pembrolizumab with 74% statistical power at one-sided type I error rate of $\alpha = 0.10$. In addition, PD-L1 status was able to impact outcomes in all lines of treatment with PD-L1+ patients having better outcomes than negative. By combining patients from DLT and the expansion phase, safety and efficacy information was available for a total of 80 patients, assuming >6 responders were observed by 40. Enrolment was not controlled for the number of patients in each line nor PD-L1 status (2nd vs. 3rd line, positive vs. negative), these numbers could vary for each patient type (eg, 2nd line PD-L1+ vs. Third line PD-L1 negative).

2. Results

2.1 Study Population

Supplementary Table S1. Patient Disposition

	Group 1<br>CPI Naïve<br>N=61	Group 2<br>CPI Relapse<br>N=19	Overall<br>N=80
	Number of patients (%)		
Screened			107
Screen Failures			27
Treated	61	19	80
Discontinued Treatment	58 (95.1)	18 (94.7)	76 (95.0)
Reason For Discontinuation of Treatment			
Progressive Disease	48 (78.7)	15 (78.9)	63 (78.8)
Adverse Event(s)	4 (6.6)	0	4 (5.0)
Patient Decision to Withdraw	1 (1.6)	0	1 (1.3)
Investigator Decision	0	1 (5.3)	1 (1.3)
Death	2 (3.3)	1 (5.3)	3 (3.8)
Other	3 (4.9)	1 (5.3)	4 (5.0)
In Treatment [1]	3	1	4
In Long Term Follow-Up [2]	9	6	15
Discontinued Study	49 (80.3)	12 (63.2)	61 (76.3)
Reason for Discontinuation of Study			
Withdrawn Consent	2 (3.3)	0	2 (2.5)
Lost to Follow-Up	1 (1.6)	0	1 (1.3)
Death	46 (75.4)	12 (63.2)	58 (72.5)

Abbreviations: CPI = Immune check point inhibitor therapy
Note: Percentages are based on the number of patients treated.
[1] Patients still on treatment at primary analysis data cut-off.
[2] Patients completed safety follow-up visit and no End of Study record.

Supplementary Table S2x. Prior Cancer Medications

ATC Class<br>Preferred term	Group 1<br>CPI Naïve<br>N=61	Group 2<br>CPI Relapse<br>N=19	Overall<br>N=80
	**Number of patients (%)**		
Any prior cancer medication [1]	61 (100)	19 (100)	80 (100)
**Other antineoplastic agents**	60 (98.4)	19 (100)	79 (98.8)
Calcium Folinate; Fluorouracil; Oxaliplatin	26 (42.6)	4 (21.1)	30 (37.5)
Oxaliplatin	17 (27.9)	10 (52.6)	27 (33.8)
Ramucirumab	10 (16.4)	6 (31.6)	16 (20.0)
Capecitabine; Oxaliplatin	10 (16.4)	2 (10.5)	12 (15.0)
Trastuzumab	8 (13.1)	3 (15.8)	11 (13.8)
Cisplatin	8 (13.1)	2 (10.5)	10 (12.5)
Nivolumab	0 (0)	8 (42.1)	8 (10.0)
Paclitaxel; Ramucirumab	4 (6.6)	2 (10.5)	6 (7.5)
Pembrolizumab	0 (0)	6 (31.6)	6 (7.5)
Durvalumab	0 (0)	4 (21.1)	4 (5.0)
Protein Kinase Inhibitors	0 (0)	2 (10.5)	2 (2.5)
**Antimetabolites**	26 (42.6)	13 (68.4)	39 (48.8)
Capecitabine	17 (27.9)	12 (63.2)	29 (36.3)
Fluorouracil Sodium	5 (8.2)	2 (10.5)	7 (8.8)
**Plant alkaloids and other natural products**	13 (21.3)	10 (52.6)	23 (28.8)
Paclitaxel	12 (19.7)	9 (47.4)	21 (26.3)

Abbreviations: ATC = Anatomical Therapeutic Chemical; CPI = Immune check point inhibitor therapy
Notes: Percentages are based on the number of patients in the Safety Population.
Medications are coded using the WHODrug Dictionary March 2019; Preferred Term = generic drug name.
If a patient had more than one medication in a drug class or preferred term, the patient was counted once in the respective drug class or preferred term.

2.2 Efficacy Outcomes

Supplementary Table S3x 2. Summary of time to Response and duration of Response – Group 1 (CPI Naïve) (Efficacy population)

	**Group 1**<br>**CPI Naïve**<br>**N=61**
Patients with response, n (%)	8 (13.1)
Time to response (months)	
n	8
Mean (standard deviation)	2.4 (1.59)
Median	1.5
Minimum, Maximum	1, 5
Responders with Events, n (%)	4 (6.6)
Responders Censored, n (%)	4 (6.6)
Data cut-off	4 (6.6)
KM Estimates of Duration (months)	
Median (95% CI)	12.5 (2.7, NE)
25th, 75th percentiles	4.2, NE
Response Duration Estimates (% of patients)	
3 Months (95% CI)	87.5 (38.7, 98.1)
6 Months (95% CI)	62.5 (22.9, 86.1)
9 Months (95% CI)	62.5 (22.9, 86.1)
12 Months (95% CI)	62.5 (22.9, 86.1)
18 Months (95% CI)	NE (NE, NE)

Abbreviations: CI = confidence interval; CPI = Immune check point inhibitor therapy; KM = Kaplan-Meier; NE = Not Estimable due to insufficient event time data

Notes: Percentages are based on the number of patients in the Efficacy Population. Duration of response was defined as the interval in months from the date measurement criteria were met for CR or PR (whichever was first recorded) until the earliest date of disease progression, as determined by Investigator assessment of objective radiographic disease assessments per RECIST version 1.1 or death due to any cause if occurring sooner than progression (date of progression – date of response / 30.4375). Patients without confirmed CR or PR were excluded from this analysis. Patients (responders) lacking evidence of disease progression, including those who initiated non-study anticancer therapy prior to disease progression, had their event times censored on the last tumor assessment date at which disease status was determined. Confidence intervals on survival estimates [S(t)] and median duration (t) calculated using complementary log-log transformation on S(t).

Supplementary table S4x 3. Summary of Overall Survival (Efficacy population)

	Group 1<br>CPI Naïve<br>N=61	Group 2<br>CPI Relapse<br>N=19
Patients with Events, n (%)	45 (73.8)	12 (63.2)
Patients Censored, n (%)	16 (26.2)	7 (36.8)
Data cut-off	14 (23.0)	7 (36.8)
Withdrawn Consent	1 (1.6)	0
Lost to Follow-up	1 (1.6)	0
KM Estimates of OS (months)		
Median (95% CI)	7.4 (5.3, 13.2)	6.5 (2.9, NE)
25th, 75th percentiles	3.8, 19.4	2.9, NE
Survival Estimates (% of patients)		
3 Months (95% CI)	81.8 (69.5, 89.5)	73.7 (47.9, 88.1)
6 Months (95% CI)	58.4 (44.9, 69.7)	52.6 (28.7, 71.9)
9 Months (95% CI)	43.4 (30.7, 55.4)	39.0 (16.8, 60.9)
12 Months (95% CI)	38.3 (26.1, 50.3)	26.0 (5.9, 52.7)
18 Months (95% CI)	25.4 (14.6, 37.7)	NE (NE, NE)
24 Months (95% CI)	14.1 (3.9, 30.7)	NE (NE, NE)
30 Months (95% CI)	NE (NE, NE)	NE (NE, NE)

Abbreviations: CI = confidence interval; CPI = Immune check point inhibitor therapy; KM = Kaplan-Meier; NE = Not Estimable due to insufficient event time data; OS = Overall survival

Notes: Percentages are based on the number of patients in the Efficacy Population. Overall survival was defined as the interval in months from the date of first dose of investigational agent until the date of death (Date of death – date of first dose + 1 / 30.4375) in Safety population. All deaths were included in the analysis. Patients who did not die had their event times censored on the latest date of last completed visit or patient contact or last known date alive. Confidence intervals on survival estimates [S(t)] and median duration (t) calculated using complementary log-log transformation on S(t).

Supplementary Table S5x 4. Summary of Progression-Survival (Efficacy population)

	Group 1<br>CPI Naïve<br>N=61	Group 2<br>CPI Relapse<br>N=19
Patients with Events, n (%)	54 (88.5)	16 (84.2)
Patients Censored, n (%)	7 (11.5)	3 (15.8)
Data cut-off	4 (6.6)	2 (10.5)
Two consecutive scans were missed prior to progression or death	3 (4.9)	1 (5.3)
KM Estimates of PFS (months)		
Median (95% CI)	1.4 (1.3, 2.5)	1.6 (1.3, 2.7)
25th, 75th percentiles	1.2, 3.6	1.3, 3.9
Survival Estimates (% of patients)		
3 Months (95% CI)	27.1 (16.6, 38.8)	28.1 (10.3, 49.2)
6 Months (95% CI)	15.3 (7.5, 25.5)	15.0 (2.9, 36.0)
9 Months (95% CI)	9.9 (3.9, 19.2)	15.0 (2.9, 36.0)
12 Months (95% CI)	9.9 (3.9, 19.2)	0.0 (NE, NE)
18 Months (95% CI)	7.4 (2.3, 16.6)	NE (NE, NE)
24 Months (95% CI)	NE (NE, NE)	NE (NE, NE)

Abbreviations: CI = confidence interval; CPI = Immune check point inhibitor therapy; KM = Kaplan-Meier; NE = Not Estimable due to insufficient event time data; PFS = Progression-free survival

Notes: Percentages are based on the number of patients in the Efficacy Population. Progression-free survival was defined as the interval in months from the date of first dose of investigational agent until the earliest date of disease progression (date of disease progression – date of first dose + 1 / 30.4375), as determined by Investigator assessment of objective radiographic disease assessments per RECIST version 1.1 or death due to any cause if occurring sooner than progression. Patients lacking evidence of disease progression, including those who initiated non-study anticancer therapy prior to disease progression, had their event times censored on the last tumor assessment date at which disease status was determined. Confidence intervals on survival estimates [S(t)] and median duration (t) calculated using complementary log-log transformation on S(t).

2.3. Safety

Supplementary Table S6x 5. Overall Summary of Treatment-Emergent Adverse Events (Safety Population)

Category	Group 1 (CPI Naïve) (n=61)	Group 2 (CPI Relapse) (n=19)	Overall (n=80)
	n (%) [Events]		
Any AE	61 (100%) [706]	19 (100%) [175]	80 (100%) [881]
Any DLT	0	0	0
**At least one AE by maximum severity[1]**			
Grade 1	3 (4.9%) [20]	4 (21.1%) [23]	7 (8.8) [43]
Grade 2	19 (31.1%) [61]	2 (10.5%) [5]	21 (26.3) [66]
Grade 3	21 (34.4%) [38]	8 (42.1%) [12]	29 (36.3) [50]
Grade 4	1 (1.6%) [1]	0	1 (1.3%) [1]
Grade 5	17 (27.9%) [17]	5 (26.3%) [5]	22 (27.5%) [22]
AE Grade 5, onset ≤30 days after last study treatment	12 (19.7%) [12]	3 (15.8%) [3]	15 (18.8%) [15]
AE Grade 5, onset >30 and ≤90 days after last study treatment	5 (8.2%) [5]	2 (10.5%) [2]	7 (8.8%) [7]
**Any AE related to bavituximab**	21 (34.4%) [60]	8 (42.1%) [23]	29 (36.3%) [83]
Any AE of severity Grade ≥3 related to bavituximab	2 (3.3%) [5]	1 (5.3%) [1]	3 (3.8%) [6]
**Any AE related to study treatment**	39 (63.9%) [126]	8 (42.1%) [31]	47 (58.8%) [157]
Any AE of severity Grade ≥3 related to study treatment	3 (4.9%) [3]	2 (10.5%) [2]	5 (6.3%) [5]
**Any AE leading to study drug withdrawn**			
Either treatment discontinuation	4 (6.6%) [4]	0	4 (5.0%) [4]
Both bavituximab and pembrolizumab discontinuation	4 (6.6%) [4]	0	4 (5.0%) [4]
**Any AE Leading to Study Drug Dose Modification**			
Either Treatment Reduced or Interrupted	28 (45.9%) [52]	5 (26.3%) [11]	33 (41.3%) [63]
Bavituximab Dose Reduced	6 (9.8%) [6]	2 (10.5%) [2]	8 (10.0%) [8]
Bavituximab Drug Interrupted, but not Pembrolizumab	11 (18.0%) [14]	1 (5.3%) [1]	12 (15.0%) [15]
Pembrolizumab Drug Interrupted, but not Bavituximab	2 (3.3%) [3]	0	2 (2.5%) [3]
Both Bavituximab and Pembrolizumab Interrupted	13 (21.3%) [29]	4 (21.1%) [8]	17 (21. %3) [37]
**Any AE Related to Study Treatment Leading to Study Drug Withdrawn**			
Either Treatment Discontinuation	1 (1.6%) [1]	0	1 (1.3%) [1]
Both Bavituximab and Pembrolizumab Discontinuation	1 (1.6%) [1]	0	1 (1.3%) [1]

Abbreviations: AE: Adverse Event; CPI: Immune check point inhibitor therapy; DLT: dose-limiting toxicity; SAE: Serious Adverse Event; n (%): Number and percentage patients in safety population with at least one event Notes: Only treatment-emergent AEs are included in summary. These are events with start date on or after the date of first dose of study treatment whose severity worsens on or after the date of first dose of study treatment. [1] Patient is counted only once at highest severity grade of all AEs experienced by the patient.

Supplementary Table S7x 6. Serious TEAEs Leading to Death by SOC and Preferred Term (Safety Population)

SOC<br>Preferred term	Group 1<br>CPI Naïve<br>N=61	Group 2<br>CPI Relapse<br>N=19	Overall<br>N=80
	Number of patients (%)		
Patients with at least one serious TEAE Leading to Death	17 (27.9)	5 (26.3)	22 (27.5)
**Gastrointestinal disorders**	1 (1.6)	0	1 (1.3)
Upper gastrointestinal haemorrhage	1 (1.6)	0	1 (1.3)
**General disorders and administration site conditions**	4 (6.6)	0	4 (5.0)
Death	2 (3.3)	0	2 (2.5)
Disease progression	1 (1.6)	0	1 (1.3)
Multiple organ dysfunction syndrome	1 (1.6)	0	1 (1.3)
**Infections and infestations**	1 (1.6)	1 (5.3)	2 (2.5)
Corona virus infection	1 (1.6)	0	1 (1.3)
Sepsis	0	1 (5.3)	1 (1.3)
**Injury, poisoning and procedural complications**	0	1 (5.3)	1 (1.3)
Injury	0	1 (5.3)	1 (1.3)
**Neoplasms benign, malignant and unspecified (incl cysts and polyps)**	9 (14.8)	3 (15.8)	12 (15.0)
Gastric cancer	7 (11.5)	3 (15.8)	10 (12.5)
Metastatic gastric cancer	1 (1.6)	0	1 (1.3)
Oesophageal cancer metastatic	1 (1.6)	0	1 (1.3)
**Respiratory, thoracic and mediastinal disorders**	2 (3.3)	0	2 (2.5)
Pneumonia aspiration	1 (1.6)	0	1 (1.3)
Respiratory failure	1 (1.6)	0	1 (1.3)

Abbreviations: CPI = Immune check point inhibitor therapy; SOC = system organ class; TEAE = treatment-emergent adverse event

Notes: Within a SOC, patients may have reported more than one preferred term. Patients are counted once for each preferred term and each SOC. Adverse events are coded using MedDRA Version 22.0.

Supplementary Table S8x 7. TEAEs Severity Grade ≥ 3 by Preferred Term in ≥ 10% Patients in Any Group (Safety Population)

Preferred Term	Group 1 CPI Naïve N=61	Group 2 CPI Relapse N=19	Overall N=80
	Number of patients (%)		
Patients with at least one TEAE	39 (63.9)	13 (68.4)	52 (65.0)
Anaemia	9 (14.8)	1 (5.3)	10 (12.5)
Gastric cancer	7 (11.5)	3 (15.8)	10 (12.5)
Abdominal pain	2 (3.3)	2 (10.5)	4 (5.0)
Blood alkaline phosphatase increased	2 (3.3)	2 (10.5)	4 (5.0)
Sepsis	0	2 (10.5)	2 (2.5)

Abbreviations: CPI = Immune check point inhibitor therapy; TEAE = treatment-emergent adverse event
Notes: Patients are counted once for each preferred term. Adverse events are coded using MedDRA Version 22.0.

Supplementary Table S9x 8. Treatment-related TEAEs by Preferred Term in ≥ 10% Patients in Any Group (Safety Population)

Preferred Term	Group 1 CPI Naïve N=61	Group 2 CPI Relapse N=19	Overall N=80
	Number of patients (%)		
Related to Bavituximab			
Patients with at least one treatment-related TEAE	21 (34.4)	8 (42.1)	29 (36.3)
Myalgia	4 (6.6)	3 (15.8)	7 (8.8)
Nausea	3 (4.9)	3 (15.8)	6 (7.5)
Headache	2 (3.3)	3 (15.8)	5 (6.3)
Related to Study Treatment			
Patients with at least one treatment-related TEAE	39 (63.9)	8 (42.1)	47 (58.8)
Fatigue	10 (16.4)	5 (26.3)	15 (18.8)
Diarrhoea	11 (18.0)	3 (15.8)	14 (17.5)
Decreased appetite	8 (13.1)	0	8 (10.0)

Abbreviations: CPI = Immune check point inhibitor therapy; TEAE = treatment-emergent adverse event
Notes: Patients are counted once for each preferred term. Adverse events are coded using MedDRA Version 22.0.

2.3 Biomarker-driven Outcomes

Supplementary Table S10: Key Efficacy Parameters by TME Panel-1 Scores and Biomarker Status (Efficacy Population) - Group 1 CPI Naïve

**Subgroup**	**n**	**ORR [1] (%)**	**DCR [2] (%)**	**mPFS [3] (95% CI)**	**mOS [4] (95% CI)**
TME Panel-1 Scores					
IA	22	22.7	45.5	1.4 (1.3, 3.9)	8.2 (3.9, 15.4)
ID	14	7.1	35.7	1.4 (1.2, 2.6)	17.7 (3.4, 20.4)
A	11	0	36.4	1.4 (1.2, 2.8)	6.3 (3.6, 13.2)
IS	10	20.0	40.0	1.6 (0.9, NE)	4.3 (1.4, NE)
Biomarker Status					
B+	32	21.9	43.8	1.5 (1.3, 3.6)	7.5 (3.9, 14.4)
B-	25	4.0	36.0	1.4 (1.2, 2.6)	6.5 (4.6, 17.7)

Abbreviations: A = angiogenic; CI = confidence interval; CR = complete response; DCR = disease control rate; IA = immune active; ID = immune desert; IS = immune suppressed; mOS = median overall survival; mPFS = median progression-free survival; NE = not estimable; ORR = objective response rate; PR = partial response; SD = stable disease

Notes: Percentages are based on the number of patients in the subgroup.

[1] The ORR was defined as the proportion of patients with a confirmed best response of CR or PR.

[2] The DCR was defined as the proportion of patients with Overall Response or with any assessment of SD, PR or CR evaluated at 6 weeks (not less than 35 days) after the first dose of study treatment.

[3] PFS was defined as the interval in months from the date of first dose of investigational agent until the earliest date of disease progression (date of disease progression – date of first dose + 1 / 30.4375), as determined by Investigator assessment of objective radiographic disease assessments per RECIST version 1.1 or death due to any cause if occurring sooner than progression.

[4] OS was defined as the interval in months from the date of first dose of investigational agent until the date of death (Date of death – date of first dose + 1 / 30.4375) in Safety population.

Supplementary Table S11: Key Efficacy Parameters by Subgroups (Efficacy Population) - Group 1 CPI Naïve

Subgroup	n	ORR [1] (%)	DCR [2] (%)	mPFS [3] (95% CI)	mOS [4] (95% CI)
NLR					
<4	39	17.9	46.2	1.8 (1.3, 2.8)	11.0 (6.4, 17.7)
≥4	22	4.5	27.3	1.3 (1.2, 1.6)	4.7 (3.0, 8.8)
Biomarker and NLR					
B+ and NLR <4	21	33.3	57.1	2.7 (1.3, 6.9)	11.0 (5.1, NE)
B+ and NLR ≥4	11	0	18.2	1.3 (0.2, 1.6)	3.6 (0.6, 8.8)
B- and NLR <4	14	0	35.7	1.4 (1.2, 2.8)	15.3 (5.8, 20.4)
B- and NLR ≥4	11	9.1	36.4	1.3 (1.2, 3.1)	4.8 (3.4, NE)
CPS					
<1	17	17.6	29.4	1.3 (1.2, 3.1)	10.0 (3.9, NE)
≥1	40	12.5	47.5	1.8 (1.3, 2.7)	6.9 (4.6, 14.4)
Biomarker and CPS					
B+ and CPS <1	5	40.0	40.0	1.4 (1.3, 13.8)	11.0 (3.9, NE)
B+ and CPS ≥1	26	19.2	46.2	1.9 (1.3, 3.6)	7.1 (3.6, 14.4)
B- and CPS <1	11	9.1	18.2	1.3 (1.0, 2.8)	10.0 (1.4, NE)
B- and CPS ≥1	13	0	53.8	2.0 (1.2, 2.8)	6.4 (3.8, 20.4)
MSI Status at Screening					
MSS	43	14.0	46.5	1.8 (1.3, 2.7)	9.1 (5.9, 17.7)
MSI unknown	18	11.1	22.2	1.4 (1.0, 1.6)	5.9 (1.5, 8.8)
Biomarker and MSI Status					
B+ and MSS	20	25.0	55.0	2.6 (1.3, 3.9)	7.6 (3.9, 19.4)
B- and MSS	21	4.8	38.1	1.4 (1.2, 2.6)	10.0 (4.6, 20.4)
B+ and MSI Unknown	12	16.7	25.0	1.4 (0.6, 6.9)	7.1 (0.6, 15.4)
B- and MSI Unknown	4	0	25.0	1.3 (0.4, 3.8)	4.8 (3.4, 15.3)
Line of therapy					
1 previous line	35	11.4	31.4	1.4 (1.2, 2.3)	7.6 (3.6, 14.9)
2 previous lines	16	6.3	50.0	2.0 (1.2, 2.8)	6.4 (4.6, 13.2)
3 previous lines	8	37.5	50.0	2.7 (1.0, NE)	NE (3.9, NE)
≥4 previous lines	2	0	50.0	2.1 (1.4, 2.7)	2.7 (1.4, 3.9)

Abbreviations: CI = confidence interval; CPI = Immune check point inhibitor therapy; CPS = combined positive score; CR = complete response; DCR = disease control rate; mOS = median overall survival; mPFS = median progression-free survival; MSI = microsatellite instability; MSS = microsatellite stability; NE = not estimable; NLR = neutrophil to lymphocyte ratio; ORR = objective response rate; PR = partial response

Notes: Percentages are based on the number of patients in the subgroup.

[1] The ORR was defined as the proportion of patients with a confirmed best response of CR or PR.

[2] The DCR was defined as the proportion of patients with Overall Response or with any assessment of SD, PR or CR evaluated at 6 weeks (not less than 35 days) after the first dose of study treatment.

[3] PFS was defined as the interval in months from the date of first dose of investigational agent until the earliest date of disease progression (date of disease progression – date of first dose + 1 / 30.4375), as determined by Investigator assessment of objective radiographic disease assessments per RECIST version 1.1 or death due to any cause if occurring sooner than progression.

[4] OS was defined as the interval in months from the date of first dose of investigational agent until the date of death (Date of death – date of first dose + 1 / 30.4375) in Safety population.

Supplementary Figure S1. Individual Neutrophils/Lymphocytes (NLR) Percent Change from Baseline by Treatment Cycle in Group 1 (CPI-naïve, Safety Population)

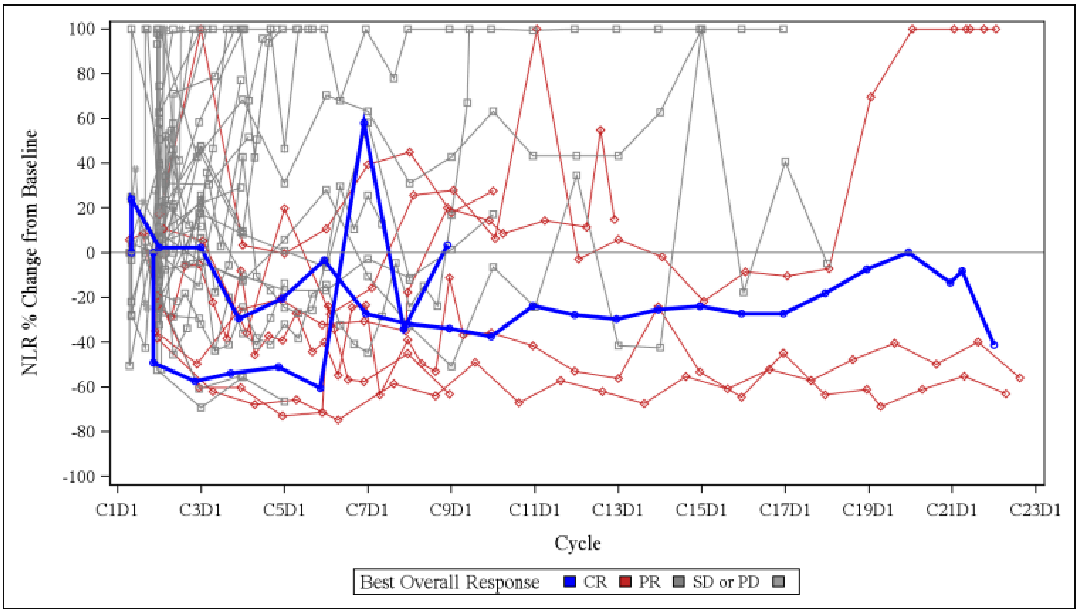

NLR dynamics (change form baseline) during treatment for each patient individually, grouped by colour for type of response. CPI-naïve population (n=61).

Supplementary Figure S2. Individual Neutrophils/Lymphocytes (NLR) Percent Change from Baseline by Treatment Cycle in Group 2 (CPI-relapse, Safety Population)

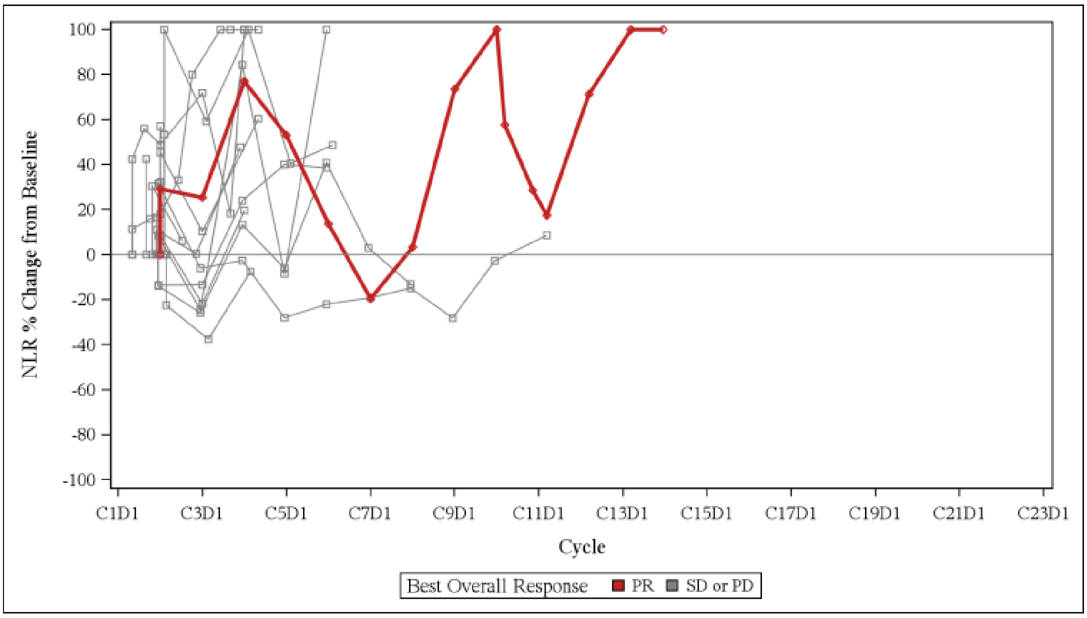

NLR dynamics (change form baseline) during treatment for each patient individually, grouped by colour for type of response. CPI-relapse population (n = 19).

2.4 Pharmacokinetic, pharmacodynamic, and immunogenicity analyses

Supplementary Figure S3: Individual Bavituximab Pharmacokinetic Concentrations Versus Treatment Cycle in Group 1 (CPI-naïve, Safety Population)

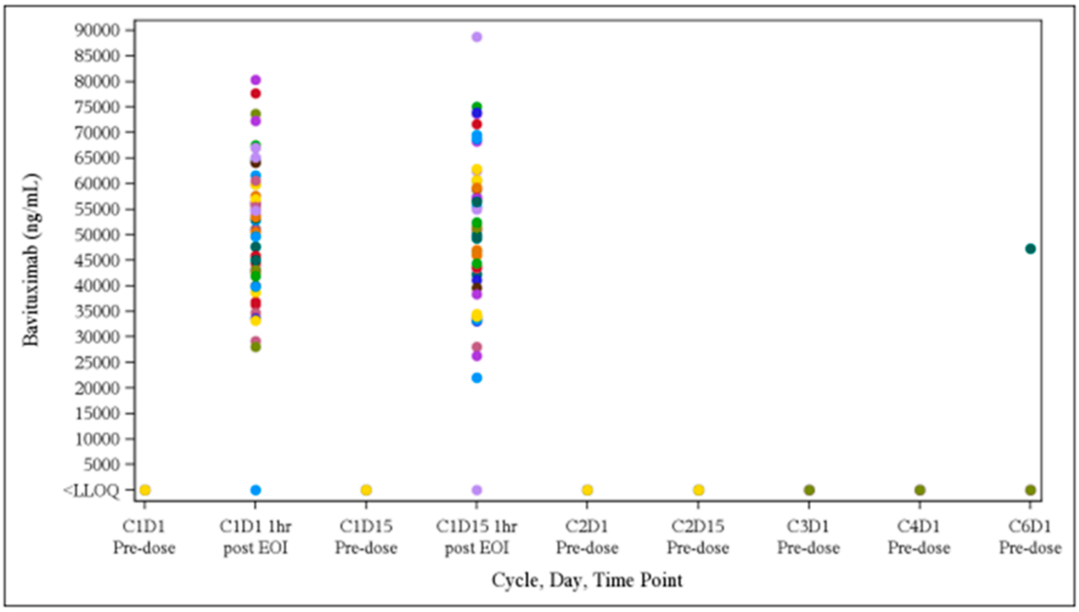

Individual Bavituximab Pharmacokinetic Concentrations Versus Treatment Cycle in Group 1 (CPS-naïve, Safety Population, n = 61)

Supplementary Figure S4: Individual Bavituximab Pharmacokinetic Concentrations Versus Treatment Cycle Safety Population in Group 2 (CPI-relapse, Safety Population)

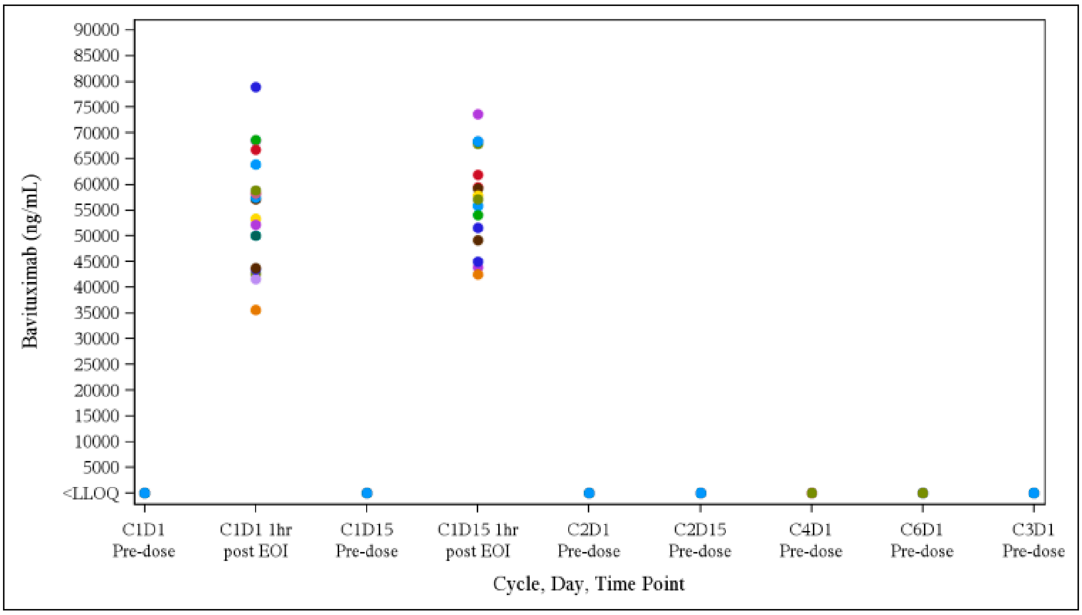

Individual Bavituximab Pharmacokinetic Concentrations Versus Treatment Cycle Safety Population in Group 2 (CPS-relapse, Safety Population, n = 19)

Supplementary table S12: Summary of Anti-Drug Antibodies (ADA) (Safety Population)

	**Group 1 CPI Naïve N=61**	**Group 2 CPI Relapse N=19**	**Overall N=80**
	**Number of patients (%)**		
Baseline ADA			
Patients missing baseline ADA	4 (6.6)	1 (5.3)	5 (6.3)
Patients with pre-existing antibodies	5 (8.2)	4 (21.1)	9 (11.3)
Patients baseline ADA negative	52 (85.2)	14 (73.7)	66 (82.5)
Overall timepoints [1]			
Patients missing post-baseline	7 (13.5)	2 (14.3)	9 (13.6)
Patients with seroconversion on treatment [2]	27 (51.9)	5 (35.7)	32 (48.5)
Cycle 2 Day 1	16 (30.8)	3 (21.4)	19 (28.8)
Cycle 4 Day 1	0	1 (7.1)	1 (1.5)
30-Day Safety Follow-up Visit	11 (21.2)	1 (7.1)	12 (18.2)
Patients remained ADA negative through all timepoints	18 (34.6)	7 (50.0)	25 (37.9)

Abbreviations: ADA = anti-drug antibodies; CPI = Immune check point inhibitor therapy
Notes:
[1] Summary of patients baseline ADA negative.
[2] Number of patients baseline ADA negative who became ADA positive for the first time.

Supplementary Figure S5: ß2-GP1 Mean Change from Baseline Concentrations Versus Treatment Cycle (Safety Population)

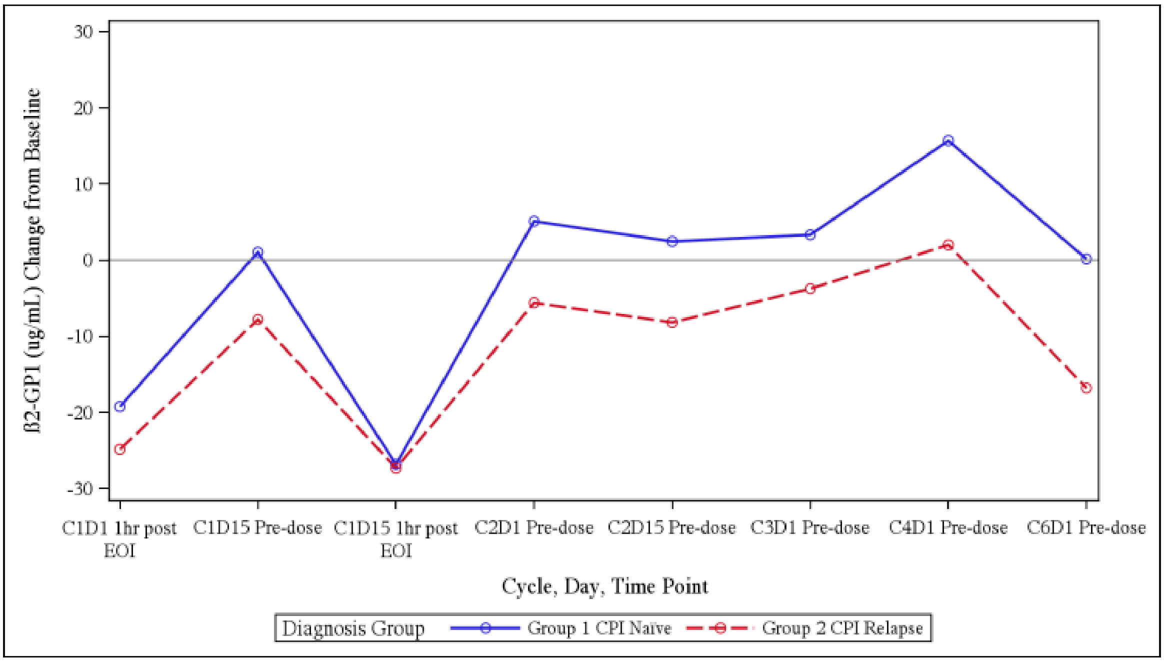

Notes: Baseline is Cycle 1, Day 1.

Mean change of β2 glycoprotein 1 (ß2GP1) from baseline concentrations across treatment cycles in the safety population. Mechanism of action of bavituximab is to form a complex of ß2GP1 along with phosphatidylserine.
